# Combining ALT/AST Values with Surgical APGAR Score Improves Prediction of Major Complications after Hepatectomy

**DOI:** 10.26502/jsr.10020179

**Published:** 2021-11-18

**Authors:** I Mitsiev, K Rubio, VP Ranvir, D Yu, AP Palanisamy, KD Chavin, I Singh

**Affiliations:** 1 Horst Schmidt Kliniken Wiesbaden, Ludwig-Erhard-Straße 100, 65199 Wiesbaden, Germany; 2 International Laboratory EPIGEN, Universidad de la Salud del Estado de Puebla, 72000 Puebla, Mexico; 3 Emmy Noether Research Group Epigenetic Machineries and Cancer, Division of Chronic Inflammation and Cancer, German Cancer Research Center (DKFZ), 69120 Heidelberg, Germany; 4 Nanjing University Medical School Affiliated Nanjing Drum Tower Hospital, Nanjing, Jiangsu, China; 5 Department of Surgery, Case Western Reserve University School of Medicine, Cleveland, OH, USA; 6 Division of Transplant and Hepatobiliary Transplant Surgery, University Hospital Cleveland Medical Center, Cleveland, OH, USA

**Keywords:** Surgical APGAR Score, Hepatectomy, Postoperative Complications, Circulating markers, Liver disease

## Abstract

Hepatectomy is a complex procedure with high morbidity and mortality. Early prediction/prevention of major complications is highly valuable for patient care. Surgical APGAR score (SAS) has been validated to predict post-surgical complications (PCs). We aimed to define a simple complications classification following hepatectomy based on a therapy-oriented severity Clavien-Dindo classification (CDC). 119 patients undergoing liver resection were included. PCs were determined at follow-up based on CDC. Clinicopathological factors were used to calculate SAS. A receiver-operator characteristic (ROC) curve analysis estimated the predictive value of SAS for PCs. Circulating markers levels of liver injury were analyzed as critical elements on PCs. SAS (P=0.008), estimated blood-loss (P=0.018) and operation time (P=0.0008) were associated with PCs. SAS was reduced in patients with (+) compared to those without (−) complications (6.64±1.84 vs 5.70±1.79, P=0.0079). The area-under-the-curve was 0.646 by ROC, indicating an acceptable discrimination with 65% possibility to distinguish (−) and (+) groups (P=0.004). Best cutoff value for SAS was ≤6/≥7, at which sensitivity and specificity were maximal. ALT/ASL levels were significantly different within the group with 9-10 SAS points (P=0.01 and 0.02). In conclusion, SAS provides accurate risk stratification for major PCs after hepatectomy, and might help improving the overall patient outcome.

## 1. Introduction

Liver resection remains the most important management in the multidisciplinary approach to most benign and malignant hepatic processes. However, hepatectomy is associated with high postoperative morbidity, high 30-day complications and high mortality [1-3]. The most characteristic complications are liver insufficiency, biliary leak and ascites [4-9]. In consequence, surgical complications have been a challenge for systematic assessment. The absence of a standard and sufficiently sensitive system to classify surgical complications has hampered proper handling of the surgical outcome. In the last decade, the Clavien-Dindo classification (CDC) [10] has been used worldwide as a standardized and validated system for the registration of surgical complications. Initially developed for reporting negative outcomes after cholecystectomy, the CDC system was modified in 2004 to increase its accuracy in clinical practice. In this system, the severity of a complication is graded based on the type of therapy required to treat the complication. Adaptations in many fields of surgery have been reported; however, the CDC system has been scarcely used in liver resection in a combined manner with other systems [11]. Thus, its applicability in this field remains unclear. Preoperative and intraoperative factors contribute to the postoperative outcome of patients who undergo major surgeries, including hepatectomy. Nationwide data collections, like the National Surgical Quality Improvement Program (NSQIP), allowed for improved preoperative risk assessment in a multitude of procedures [12]. Research has been done to identify preoperative conditions which could be used to assess the success of liver resections and to create a scoring system for outcome prediction [13,14]. Until the introduction of the Surgical APGAR (Appearance, Pulse, Grimace, Activity, and Respiration) Score (SAS) [15] there has been a scarcity of easy to use risk assessments of intraoperative parameters. For instance, POSSUM [16] is a complex score using multiple variables. Similarly, the APACHE score [17] has been adapted for using intraoperative data but displays difficulties to calculate and to use in clinical routine. SAS has been designed to be a simple, readily available tool, using only three intraoperative objective variables for predicting postoperative outcome: estimated blood loss (EBL), lowest heart rate (HRlow), and lowest mean arterial pressure (MAPlow) during the surgery.

Many groups have previously demonstrated that SAS is a useful model to predict early complications after a variety of procedures, such as radical cystectomy, colon and rectal resection, herniorraphy, thyroidectomy, parathyroidectomy, endovascular repair of abdominal aortic aneurysm, cytoreduction, esophageal resection, gastrectomy, etc. [18-22]. It was also validated globally in a multicenter clinical investigation in 8 countries [23], and also in the setting of Electronic Medical Recording [24], which was originally thought to be a potential obstacle in obtaining valid calculations. Moreover, SAS has been recently found to be a useful tool for predicting postoperative complications after hepatectomy for hepatocellular carcinoma [25]. However, to the best of our knowledge, SAS has not been investigated for predicting postoperative complications in hepatectomy for both benign and malignant indications.

## 2. Methods

### 2.1 Basic patient information

Out of 145 patients who met inclusion criteria, 26 had missing or incomplete anesthesia records. A total of 119 patients (41 men, 78 women; mean age: 50.48 years) undergoing liver resection from January 1^st^ 2002 until January 1^st^ 2012, in the department for Hepatobiliary and Transplant Surgery of the Medical University of South Carolina, USA, were identified. Thirteen patients had MELD score ≥ 9 (9-15). Forty-one patients underwent ≤ 2 segment hepatic resections, and seventy-eight underwent > 2 segment hepatic resections. Liver resection was defined as partial resection of hepatic tissue for the treatment of hepatic diseases, excluding liver transplantation or segmental liver procurement for living donor transplantation. Postoperative complications were determined by the surgeon team in the course of the hospital stay or at a postoperative presentation, based on the Clavien-Dindo classification system (CDC). Clinicopathological parameters, including lowest mean arterial pressure, lowest heart rate, and estimated blood loss, were gathered and used to calculate SAS points (Table 2). We used a retrospective analysis of a prospectively maintained database with the IRB approval number Pro00017832 of the institutional review board of the Medical University of South Carolina, USA.

### 2.2 Selection of predictors of major complications

Patient data collected included demographics, operative procedure and indications for surgery, anesthetic approach, intraoperative physiologic data, and outcomes before discharge from the hospital. A total of 132 variables were extracted from the electronic information management system. The following preoperative variables were collected: demographics, diagnosis, cancer stage, liver function, coagulation function, hepatitis serology data, comorbidities, and a routine blood test results. We collected additional 28 variables from the intraoperative anesthesia records including highest systolic blood pressure, highest diastolic blood pressure, MAP (initial, final, highest, and lowest), heart rate (initial, final, highest, and lowest), central vein pressure (initial, final, highest, and lowest), estimated blood loss (EBL), operation routine and protocol, operative duration (incision-to-skin closure time), temperature and oxygen saturation (initial, final, highest, and lowest), volume of urine output, volume of IV fluids with colloid, crystalloid, and blood products administered, use of pressure support medication, and anesthesia type and time. Hemodynamic variables were recorded every 5 minutes, and other variables were updated hourly. The complications mentioned below were also collected.

### 2.3 Definition of major complications after liver resection

Dependent variables related with outcomes were mortalities or morbidities within 30 days after operation. The following events were defined as major complications; acute renal failure, bleeding requiring ≥ 4 U red cell transfusion within 72 hours after operation, cardiac arrest requiring CPR, coma for 24 hours or longer (intubation greater than 24 hours), deep venous thrombosis, septic shock, myocardial infarction, unplanned intubation, ventilator use for 48 hours or longer, pneumonia, pulmonary embolism, stroke, wound disruption, deep or organ-space surgical site infection, sepsis, systemic inflammatory response syndrome, and vascular graft failure, according to NSQIP established definitions. A group of physician reviewers determined by consensus the following special major complications after liver resection: biliary fistula or bleeding requiring further operation and acute hepatic failure requiring hepatic support or liver transplantation. Patients having complications categorized in the database as “other occurrence” were reviewed individually, and the severity of the occurrence was evaluated according to the CDC system. The complications of CDC Class III and greater (requiring surgical, endoscopic, or radiologic intervention or intensive care admission; or life-threatening) were also considered as major complications.

### 2.4 Statistics

Univariate analysis was performed between each preoperative and intraoperative variable with major complications using Pearson’s Chi-square for nominal variables or Student t test for continuous variables. Complication rates by individual and categorized SAS or CDC were compared using Chi-square test. Patients with SAS 9-10 served as reference. The discrimination was computed with the c-statistic from a univariable logistic regression using SAS as a categorical predictor, and the incidence of major complications as the outcome. SAS statistical software version 9.1 (SAS institute, Cary, NC) was used to analyze all data. To access the potential of the SAS points system to predict major complications, receiver operator characteristic (ROC) analysis was performed and the area under the curve (AUC) was analyzed according to DeLong et al [26]. The value of AUC was defined from 0.5 as no discrimination to 1.0 as perfect discrimination. A value of 0.6-0.7 was considered as moderate, 0.7-0.8 as reasonable, and over 0.8 as good. Data were represented as mean ± standard deviation and the difference between groups were assessed by student’s T-test. All statistical analyses were performed with GraphPad Prism, version 9.0.0. P-value<0.05 was considered statically significant [27-31].

## 3. Results

### 3.1 Incidence of postoperative complications

The clinicopathological preoperative and intraoperative characteristics of all 119 patients (41 male, 78 female, average age 50.48 ± 15.93 years old) are summarized in Table 1. In turn, patients were categorized as “All patients”, “Patients without complications” and “Patients with complications”. The operation time was 216.18 ± 90.29 min, the estimated blood loss (EBL) 744.96 ± 971.31 mL, the lowest heart rate 65.31 ± 11.90 beats/min, and lowest mean arterial pressure 63.34 ± 8.35 mmHg. Nonanatomical and anatomical resections were performed in 6 (5.04%) and 113 (94.96%) patients, respectively. Postoperative complications were rated as grade >3 (P=0.0014) by the Clavien-Dindo scores. Major postoperative complications were observed in 45 patients (37.82%). The complication with the highest occurrence was biliary leak (17.64%). Eight patients underwent reoperation secondary to biliary leak, bleeding, hematoma, or wound dehiscence. Four patients died on days 27, 31, or 35 after liver resection with portal vein thrombosis, hepatic artery thrombosis, and multiorgan system failure, respectively. Hospital mortality was 3.36%. Pleural effusion (23.35%) and atelectasis (20.17%) were most common overall complications. Patients with complications were an average of 10 years older (57.01 versus 46.6, P=0.0004) than patients without complications. Men represented 34.45% of all patients (41/119) undergoing liver resection, but they comprised 51.1% of all patients (23/45) with complications. Patients with more comorbidities had higher rates of major complications compared to patients with less comorbidities (P=0.007), based on the Carlson index. In addition, patients with malignancies and high bilirubin levels had a higher rate of major complications (P=0.0013 and P=0.0202). Furthermore, hepatitis history, height and ethnicity were also associated with postoperative major complications (P=0.0137, P=0.0015 and P=5E-42). However, comorbidities, such as cirrhosis, preexisting pulmonary disease, hypertension, or diabetes mellitus, were not associated with postoperative adverse events. Body mass index and medical history, such as smoking, alcohol, or drug usage history, were not significantly associated with complications [32-35].

### 3.2 Relationship between SAS and postoperative complications

**Figure 1:**
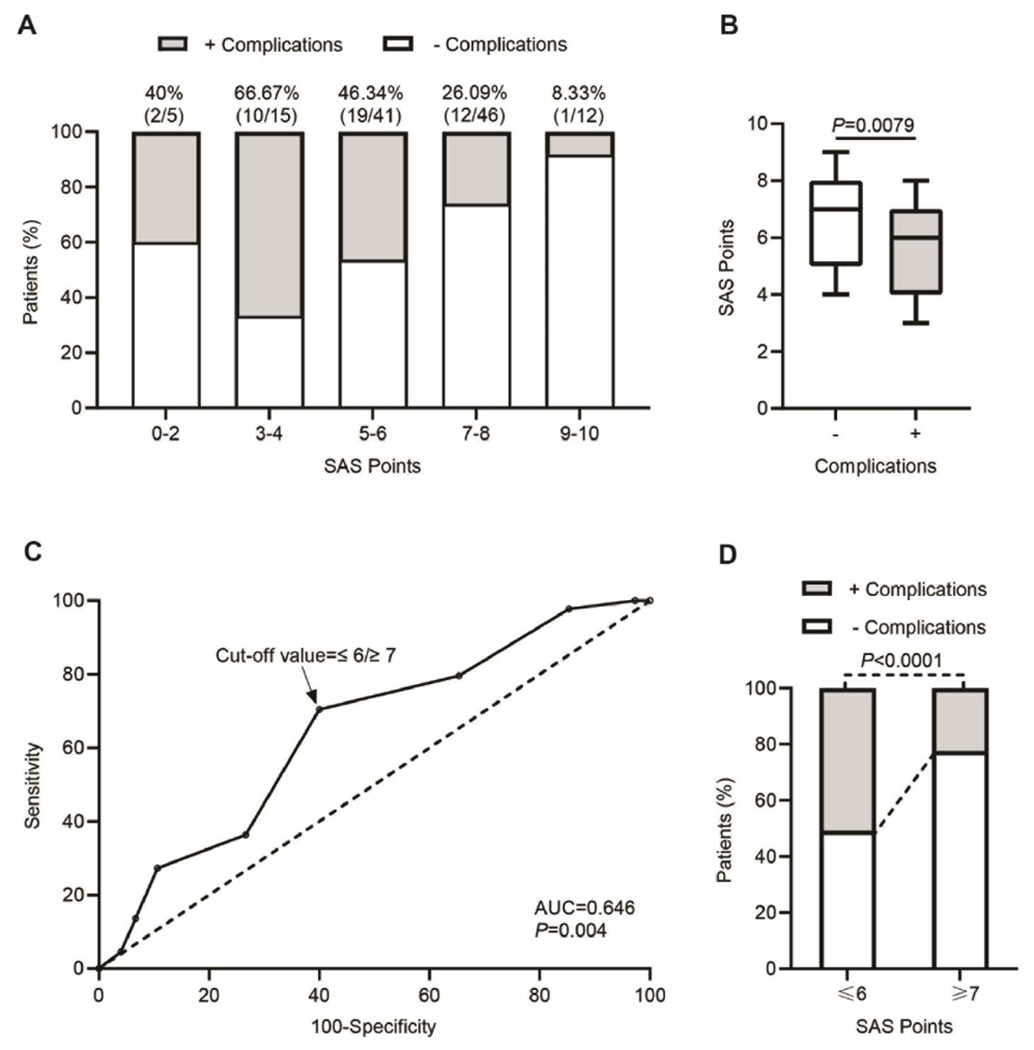
Relationship between SAS and postoperative complications. A) Distribution of patients with and without complications across different range of SAS points (0-2, 3-4, 5-6, 7-8, 9-10). Height of the bar indicates percentage of patients. The percentage above bars indicates the ratio of patients with complications after hepatectomy. B) Box and whisker plot representing SAS points of the patients without (−) or with (+) complications after hepatectomy, in which the lines within the box represent median value; the upper and lower lines of the boxes represent the 25^th^ and 75^th^ percentiles respectively; and the upper and lower bars outside box represents the 95^th^ and 5^th^ percentiles respectively. C) Receiver operator characteristic (ROC) curve of SAS points for predicting development of complications after hepatectomy. The calculated area under the curve (AUC) is shown on the graph and the dotted line indicates the best cutoff values (≤6/≥7) with maximum sensitivity and specificity. D) Bar plot representing percentage of patients without (−) or with (+) complications across SAS points cutoff values (≤6/≥7) after hepatectomy.

The surgical APGAR score (SAS) was calculated using three intraoperative variables (Table 2): estimated blood loss (mL), lowest mean arterial pressure (mmHg) and lowest heart rate (beats/min). One out of three intraoperative variables was independently associated with major complications (estimated amount of blood loss, P=0.018). The distribution of the SAS scores for all patients without (−) and with (+) complications after liver resection is shown in Figure 1A, across a different range of SAS points (0-2, 3-4, 5-6, 7-8, 9-10). The proportion of cases with postoperative complication in groups 0-2, 3-4 and 5-6 was higher (40%, 66.67% and 46.34%, respectively) compared to groups 7-8 and 9-10 (26.09% and 8.33%, respectively). The total SAS points between the group of patients (−) and (+) was significantly different, the mean for the total number of patients, patients (−) and patients (+) was 6.29 ± 1.87, 6.64 ± 1.84 and 5.70 ± 1.79, respectively. The median for patients (−) and patients (+) was 7 and 6, respectively (P=0.0079, n=75 and n=44, respectively, Figure 1B). In order to estimate the predictive value of the SAS score for the possibility of postoperative complications, we performed the receiver-operator characteristic (ROC) curve analysis. The calculated area under the curve (AUC) was 0.646, which indicates an acceptable discrimination with a possibility of 65% that the predictive model will be able to distinguish between the (−) and (+) groups (P=0.004, Figure 1C). In addition, the ROC analysis showed a best cutoff value for the SAS score ≤6/≥7, at which the sensitivity and specificity values of the model were maximal (for ≤6, sensitivity = 0.70 and specificity = 0.60; for ≤7, sensitivity = 0.80 and specificity = 0.35; Figure 1C). Patients with SAS points ≤6 displayed a significantly higher incidence (P=0.0001) of postoperative complications (31/61 patients, 50.82%) than those with ≥7 points (13/58 patients, 22.41%, Figure 1D), with mean values of 4.84 ± 1.32 and 7.77 ± 0.58, and median values of 5 and 8, respectively.

### 3.3 Increased serum ALT/AST levels as a potential biomarker for postoperative complications

**Figure 2:**
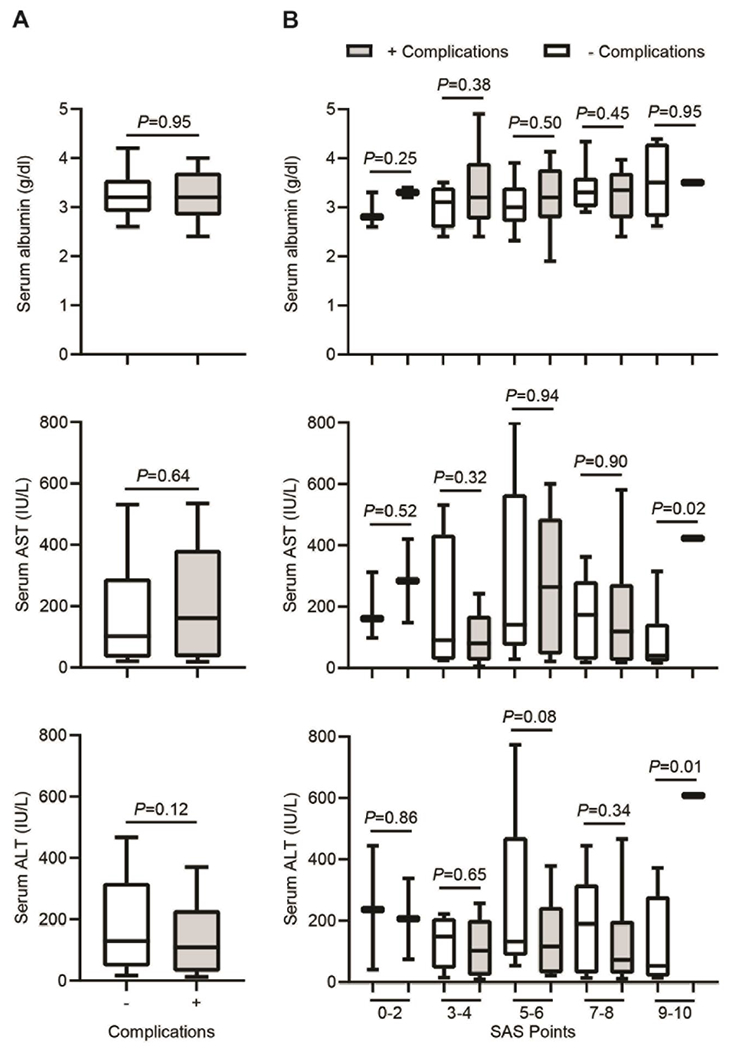
Increased serum ALT/AST levels as a potential biomarker for postoperative complications. A) Box and whisker plot representing serum albumin, serum AST and serum ALT levels across patients without (−) and with (+) complications after hepatectomy. B) Box and whisker plot representing serum albumin, serum AST and serum ALT levels across different range of SAS points (0-2, 3-4, 5-6, 7-8, 9-10) after hepatectomy. In both plots, the lines within the box represent median value; the upper and lower lines of the boxes represent the 25^th^ and 75^th^ percentiles respectively; and the upper and lower bars outside box represent the 95^th^ and 5^th^ percentiles respectively.

To determine the contribution of diverse clinicopathological factors, we compared the patients without (−) and with (+) postoperative complications according to the preoperative and intraoperative characteristics included in Table 1. The mean serum albumin levels were not significantly different between both groups (3.28 ± 0.57 g/dl in patients (−) compared to 3.27 ± 0.65 g/dl in patients (+), P=0.95, Figure 2A, top). No significant differences in this circulating marker of liver injury were detected upon a patient classification across a different range of SAS points as in Figure 1, for any of the groups (Figure 2B, top). Among the most sensitive and widely used liver enzymes detected in serum upon liver injury are the aminotransferases, including aspartate aminotransferase (AST or SGOT) and alanine aminotransferase (ALT or SGPT). Interestingly, despite no apparent differences in the mean values of serum AST between the total number of both groups of patients (195.38 ± 220.75 IU/L in patients (−) compared to 215.25 ± 201.12 IU/L in patients (+), P=0.64, Figure 2A, middle), a significant difference is observed between patients included in the group with SAS points 9-10 (96.2 ± 103.25 IU/L in patients (−) compared to 423 ± 423 IU/L in patients (+), P=0.02, Figure 2B, middle). Furthermore, significant differences are not observed in the mean values of serum ALT between the total number of patients in both groups (209.39 ± 217.56 IU/L in patients (−) compared to 149.55 ± 144.58 IU/L in patients (+), P=0.12, Figure 2A, bottom). Presumably, a significant difference arises between both groups of patients included in the group with SAS points 9-10 (127.4 ± 133.32 IU/L in patients (−) compared to 608 ± 608 IU/L in patients (+), P=0.01, Figure 2B, bottom).

## 4. Discussion

Our work demonstrates a good performance of the surgical APGAR Score (SAS) for predicting postoperative complications after liver resection in patients either with hepatocellular carcinoma (HCC) or other benign/malign etiologies. To date, few groups have demonstrated the usefulness of the SAP score calculation in HCC patients with preoperative, but not intraoperative parameters. We detected novel parameters significantly altered in patients with postoperative complications compared to patients without complications. Thus, we propose for the first time the inclusion of two circulating markers in patients with high SAS points, which might suggest, upon further robust validation, an independent early-parameter positively correlating the SAS score prediction.

Preoperative and intraoperative factors have significant contributions to the postoperative outcome of patients who undergo major surgeries including hepatectomy [36,37]. Hepatectomy is a major surgery for patients with highly compromised liver disease and comorbid conditions associated to liver injury [38]. This intervention is associated with a high risk of postoperative complications and morbidity [39]. Patients undergoing liver resection are by routine monitored in intensive care units and harbor a high risk of perihospital complications [40]. Early identification of high-risk and low-risk patients would be overall beneficial in clinical practice. Public data collections, like the National Surgical Quality Improvement Program (NSQIP), were stablished by the American College or Surgeons with the purpose to improve the preoperative risk assessment [41]. Despite such concerns, the transition on the eligibility from preoperative to intraoperative parameters was itself challenging and innovative. On the contrary to the predictors of initial use, the SAS score is a simple, straightforward, rapid 10 point-scoring method integrated by the contribution of 3 parameters extracted from anesthesia records: estimated blood loss, lowest intraoperative heart rate and lowest intraoperative mean arterial pressure during the surgical intervention [42]. One clear advantage is that the SAS method does not require elaborated calculations. Previous groups have added considerable amount of evidence for the efficient predictive use of the SAS score in multicenter studies with general surgical patients [43-45], and in a relevant manner on interventions involving resections of esophagus, stomach, colon, radical cystectomy, thyroid, and aorta [46,19,47,48]. Before the introduction of the SAS score by Gawande et. al. in 2007 [15], the risk assessment in HCC patients undergoing liver resection was rather uneasy due to the introduction of multiple heterogeneous variables [27, 28], and their applications in clinical schedules. The efficiency of the SAS score for liver surgeries was first validated by Pearson et al [29] in 2017, at a later time-point compared to the establishment of SAS score for other surgical interventions, due to the concerns on blood loss usually exceeding the highest EBL category. Previous predictors include the score for end-stage liver disease (MELD), commonly used as a predictor for liver pre-transplant mortality [30,31]. The first use of the surgical Apgar score using strictly intraoperative variables was first validated in 2017 [29]. Since then, abdominal and vascular surgeries have rendered similar approximations [32-35]. Importantly, we observed that the levels of the circulating markers of liver injury are a critical element on postoperative predictions following liver resection. In our cohort, only alanine aminotransferases (ALT) and aspartate aminotransferase (AST) turned to be significantly different between the two groups of patients (without and with complications) scored with SAS points 9-10, but we did not detect differences for serum albumin. Our patient population is not large enough to draw any firm conclusions about this topic. Nevertheless, our results are encouraging for further research in this direction, despite nor being consistent with Tomimaru et al [25]. Several differences might arise from the exclusive use of HCC patients versus the recruitment of patients with diverse highly compromised liver diseases. In addition, our AUC seemed slightly lower compared to the one calculated previously in HCC patients. We obtained a cut off value <6/>7, a similar value to the obtained by other cohort with HCC [25]. Indeed, a common observation by different groups is that a cut off value of 6/7 points is optimal to differentiate between low- and high-risk patients [49]. This reflects that despite individual differences in the preoperative and intraoperative parameters, the SAS predictive efficiency harbors comparable sensibility and specificity values among dissimilar populations. Some limitations of our study include the fact that the cohort was recruited from a single institution with the participation of a limited number of surgeons. However, the analysis was lead by two members of the surgical team in an unbiased patient-anonymized manner. Also, the intrinsic variability among patients in the parameters used to calculate the SAS score, naturally expected, is clearly reflected on the wide standard deviations included in Table 2. Despite these limitations, this is the first study in which additional intraoperative parameters are significantly different and associated with postoperative complications. Most of the clinical studies carried on until now aim to compare the AUC values between their internal predictors and the SAS score. With our study, we add to the contributions of rather few approaches including intraoperative, and not only preoperative, parameters for the statistical analysis.

## Figures and Tables

**Table 1:** Preoperative and intraoperative characteristics of patients with and without major complications.

	All Patients	Patients without major Complications	Patients with major Complications	P-value
**N**	119	75	44	
**Preoperative characteristics**				
Gender (M/F)	41/78	18/57	23/21	0.4999
Age (years)	50.48±15.93 n=118	46.59±14.51 n=74	57.01±16.24	0.0004
Height (cm)	167.81±12.38 n=118	165.07±12.33 n=74	172.43±11.15	0.0015
Ethnicity (AA/C/A/H)	44/71/2/2	26/47/2/0	18/24/0/2	5.00E-42
Weight (kg)	80.04±24.90 n=118	77.94±19.1 n=74	83.57±32.32	0.2362
ASA (1/2/3)	14/47/58	8/33/34	6/14/24	2.30E-06
HAV (+/−)	2/117	0/75	2/42	0.5
HBV (+/−)	4/115	2/73		0.5
HCV (+/−)	11/108	3/72		0.5
Platelets (K/mm3)	212.99±86.44	215.59±78.14	208.57±99.81	0.6708
PT (%)	15.85±2.45 n=90	15.50±2.19 n=51	16.29±2.72 n=39	0.1284
T-Bilirubin (mg/dl)	1.80±2.15 n=109	1.43±1.09 n=68	2.41±3.14 n=41	0.0202
Albumin (g/dl)	3.27±0.60 n=109	3.28±0.57 n=69	3.27±0.65 n=40	0.9477
AST (U/L)	202.67±213.03 n=109	195.38±220.75 n=69	215.25±201.12 n=40	0.6409
ALT (U/L)	187.43±195.42 n=109	209.39±217.56 n=69	149.55±144.58 n=40	0.1238
Liver cirrhosis (+/−)	10/109	3/72	7/37	0.5
**Intraoperative characteristics**				
Operation Time (min)	216.18±90.29 n=116	195.08 ±73.49 n=73	252.00±104.76 n=43	0.0008
EBL (ml)	744.96±971.31	584.67±734.28	1018.18±1240.92	0.0181
Lowest mean arterial pressure (mmHg)	63.34±8.33 n=117	63.30±7.99	64.94±9.09 n=41	0.9532
Highest mean arterial pressure (mmHg)	104.74±15.39 n=117	103.41±14.28	109.72±17.32 n=41	0.214
Lowest heart rate (per min)	65.31±11.90	63.81±11.30	67.86±12.58	0.0729
Highest heart rate (per min)	100.08±17.28	98.45±16.89	102.84±17.78	0.1823
SAS (points)	6.29±1.87	6.64±1.84	5.71±1.79	0.0078
Resection Type (nonanatomical/anatomical)	6/113	5/70	1/43	0.5
Laparoscopy (+/−)	33/86	27/48	6/38	0.5
Etiology (malignant/benign)	48/71	20/55	28/16	0.4999

AA = African-American; C = Caucasian; A = Asian; H = Hispanic; ASA = American Society of Anesthesiologists; HAV = hepatitis A virus infection; HBV = hepatitis B virus infection; HCV = hepatitis C virus infection; PT = prothrombin time; T-Bilirubin = total bilirubin; AST = aspartate transaminase; ALT = alanine transaminase; EBL = estimated blood loss; SAS = surgical APGAR score. Data are expressed as number of patients or mean ± standard deviation.

**Table 2:** Surgical APGAR Score.

Variables	0 Points*	1 Point	2 Points	3 Points	4 Points
Estimated blood loss (mL)	> 1000	601 – 1000	101 – 600	≤ 100	
Lowest mean arterial pressure (mmHg)	< 40	40 – 54	55 – 69	≥ 70	
Lowest heart rate (beats/min)	> 85	76 – 85	66 – 75	56 – 65	≤ 55

* Occurrence of pathological bradyarrhythmia (including sinus arrest, atrioventricular block of dissociation, junctional or ventricular escape rhythms) and asystole also receives 0 points for lowest heart rate. Highest score is 10 and correlates with a good outcome. Lowest score is 0 and is associated with a worse outcome.
